# A comparison of box-to-box and positional possession styles: selecting an appropriate game format for U13 footballers (a pilot study)

**DOI:** 10.3389/fspor.2025.1634446

**Published:** 2025-10-02

**Authors:** Imants Bleidelis, Signe Luika, Aleksandrs Kolesovs, Behnam Boobani

**Affiliations:** ^1^ Riga Stradins University, Latvian Academy of Sport Education, Riga, Latvia; ^2^ Department of Psychology, The University of Latvia, Riga, Latvia

**Keywords:** tactical behavior, field zone, player's effectiveness, football, youth

## Abstract

**Introduction:**

This pilot study aimed to examine trends in the use of game zones, group interactions of players participating in a game episode, and the effectiveness of their actions in the completion zone for the age group U13, between the game formats 9 vs. 9 and 11 vs. 11.

**Methods:**

Latvian Football Players (*n* = 45), six goalkeepers and 39 field players, 12.6 ± 0.7 years of age; height: 1.65 ± 0.18 m; weight: 54.3 ± 10.2 kg; BMI: 26.7 ± 3, in three teams (from Riga), played two one-day tournaments (game order: C-B, C-A, and B-A) using two competition formats. A quasi-experimental design was used to assess the tendencies of changes in game format. Observational methodologies were used to analyze the six match recordings by the Lince 2.1 computer program, recorded with two VEO Gen.2 cameras, to evaluate the start and end zones of game episodes, including shots at the opponent's goal.

**Results:**

It was found that when choosing between the formats 9 vs. 9 and 11 vs. 11 in the U13 age group, it is possible to expect visible change (based on a medium effect size) in distribution of start and end of game episodes with more often reaching the completion zone, (the 1/3 area of the field where the opponent's goal was located), increasing number of players involved in the game episode, and the increasing number of cases of changing the direction of the attack under 9 vs. 9 format.

**Conclusion:**

The results showed that when choosing the format of youth championships, coaches and stakeholders should consider its impact on the formation of game patterns in players.

## 1 Introduction

Research in the field of youth Football players has been ongoing for many years (1). Changing the game format from 11 vs. 11 to a smaller player format can be categorized as a football sided game (2), which can be divided as small sided games (SSG 2 vs. 2, 3 vs. 3, 4 vs. 4), medium sided games (MSG—5 vs. 5, 6 vs. 6, 7 vs. 7), and large sided games (LSG—8 vs. 8, 9 vs. 9, 10 vs. 10) (3). These types of game formats widely used as a training suitable for players with different skill (4), reduction in the number of players, the playing field is scaled down (5, 6), multiple rule modifications are implemented (7), and the format of the game is more adapted to the physical and psychological development of players (8).

According to the Survey of the Union of European Football Associations (UEFA), the main formats of play at the age of U13 are 9 vs. 9 and 11 vs. 11. Changing the rules of play, the competition, and the competitive environment makes an impact on the technical and tactical skills of the players and on their development (9).

While 11 vs. 11 places higher demands on the physical fitness of players, associated with an increase in the relative field area per player (10), with a clear definition of the tactical role of players on the field (11, 12), it raises concerns about the development of individual and group interactions between players (13), their willingness to use tactical techniques from adult football (14), and influence on the development of personalized approaches in youth formats (15).

Also, the rules and format of competition in youth sports must be adapted to the needs of children to provide them with the appropriate environment (16), many authors conclude that 11 vs. 11 game formats, even on a field with the smallest possible dimensions (17), is too large for players until the U13 age group (18). They spend too much time without the ball and have fewer opportunities for passing interactions, as well as limited chances for tactical skill and individual technical improvement (19).

Moreover the 9 vs. 9 is more in line with player physiological needs (20), suitable for developing individual technique (21), the player demonstrates greater control over the playing space (22), the number of technical actions and scanning activity of players increases (23). Without taking into account the influence of the game context of a football match (24) and focusing mainly on the frequency of game actions (25), which only serves as evidence of more frequent turnover of teams ball possession (26) further research is needed to investigate the use of game zones in 9 vs. 9 and 11vs 11, players involved in the game episode, and the effectiveness of their actions. Taking into account the mentioned considerations, this study aims to investigate the player interaction in the U13 age group in two different game formats, 9 vs. 9 and 11 vs. 11, by analyzing game zones, group interactions of players participating in a game episode, and the effectiveness of their tactical actions in the completion zone for the development of players' skills in the context of youth competitions. Therefore, the research question is: How do player interactions and tactical effectiveness vary between 9 vs. 9 and 11 vs. 11 formats in U13 youth football? Also, it is hypothesized that U13 players in the 9 vs. 9 format will show higher interaction rates and more effective tactical actions in the completion zone compared to those in the 11 vs. 11 format.

## 2 Methodology

### 2.1 Participants

Teams and participants were selected based on judgmental sampling (27), in which the researcher used his own choice to select participants for inclusion in the study. This selection is not meant to represent the general population. It may introduce bias into the study, but it offers a chance to identify trends, develop a new approach, and gather additional data to expand on existing information (28).

Three teams (*n* = 45 players) with the highest status were selected from the Latvian Football Federation, A-level academies from the Riga region, 6 goalkeepers and 39 field players, 12.6 ± 0.7 years of age; height: 1.65 ± 0.18 m; weight: 54.3 ± 10.2 kg; BMI: 26.7 ± 3), who participated in the 9 vs. 9without any experience in the 11 vs. 11 format. One week before the start of the Latvian Football Federation Youth Championship in the Elite U13 group, a round-robin tournament was organized for three approximately equal teams (game order: C-B, C-A, B-A), which each team played one game in the same format against the other team (29). Each team participated in the tournament, first playing in a 9 vs. 9 game format, and then in a tournament where the game format was changed to the 11 vs. 11 format. After the 9 vs. 9 competition, a rest day was provided before the 11 vs. 11 tournament to avoid possible accumulated fatigue that interfered with the quasi-experiment.

The total number of games played in both tournaments was *n* = 6, with three games played in the 9 vs. 9 format and three games in the 11 vs. 11 format and game duration 30 min. Between games, players were given a 15-minute rest period in which to recover before the next game. To preserve the spontaneity of the subjects' reactions to the ongoing events, all matches had the character of friendly games and coaches were forbidden from changing the tactical plan of the players and demanding a specific result from the game.

### 2.2 Study design

A quasi-experiment used an observational methodology (30), which is defined as a procedure aimed at structuring the conscious perception of manifested reality, with its correct interpretation. To obtain more accurate results using observational methodology, additional requirements were met (31), including spontaneity of behavior. The study was carried out in a natural context, in small groups of people who have a connection with each other (one team).

### 2.3 Procedures

**Figure 1:**
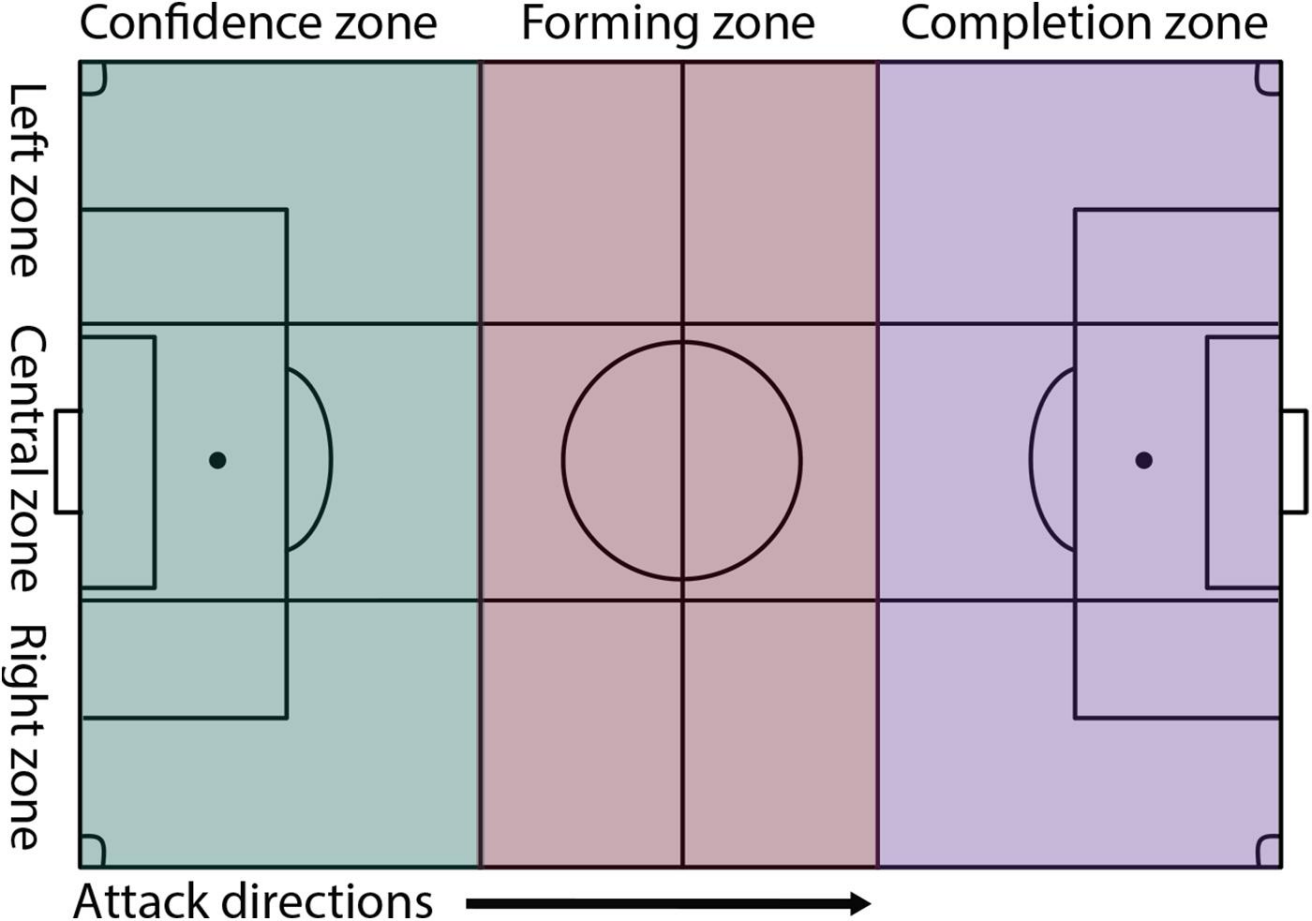
Divided football field.

The football field was divided transversely and longitudinally into three equal parts (32). This created nine identical game zones (Figure 1), as the defensive zone (the 1/3 area of the field where the team's own goal was located).the forming zone (the 1/3 area of the field located in central zone of the pitch) and the completion zone (the 1/3 area of the field where the opponent's goal was located). The following conditions were also ensured that the start of the game on match days was at the same time; participating teams played with the same players for two tournaments; coaches did not disrupt the game by only making substitutions between games or in cases where the player could not continue the game due to injury or fatigue; normal offside rules the same for both formats; the same referee officiated the all the matches; ball size No. 4.; the goal size 2.44 m × 7.32 m. The area of the football field was 74 m × 52 m, for the 9 vs. 9 format (214 m^2^ relative field width per player); the size of the football field for the 11 vs. 11 format was 106 m × 68 m (328 m^2^ relative field width per player).

The longitudinal zones of the observed team have the following names: right zone, central zone, left zone.

The transverse zone sizes varied depending on the game formats:

• 24.7 m × 17.3 m, game format 9 vs. 9;
• 35.3 m × 22.7 m, game format 11 vs. 11.

In addition, the forming zones (central zone) were divided into two sub-zones, each of which started from the center line of the field:

• 12.35 m × 17.3 m, game format 9 vs. 9;
• 17.65 m × 22.7 m, game format 11 vs. 11.

Accordingly, the sizes of the longitudinal zones varied depending on the game formats:

• 74 m × 17.3 m, game format 9 vs. 9;
• 106 m × 22.7 m, game format 11 vs. 11.

All data for the study were collected based on analysis of game recordings. Six games were filmed with two VEO Gen.2 cameras (33) placed on tripods (5.2 m and 7.4 m) in the center of the playing field. After filming, the video from the cameras was uploaded to the VEO online server and then processed and downloaded to a computer for further encoding. Two teams were analyzed in each game. In total, the actions of 12 teams were analysed in six games.

The main data processing device was a computer with an AMD Ryzen 9 5900X 12 core processor (3.70 GHz), 32 GB of RAM, Windows 11 PRO 64-bit operating system, and a Samsung SSD 980 PRO 1 TB NVMe drive. The software application Lince version 1.2.1 (34) was deployed on the central computing unit to facilitate the coding of data. This program offers comprehensive functionalities to oversee a wide range of events, systematically encode them, and subsequently enable the exportation of the results in multiple data formats.

Each game consists of a series of game episodes, which are divided into a set of technical-tactical actions and other elements, which were coded accordingly. The observation sample in the 9 vs. 9 format consists of 190 game episodes, in which the zones of the beginning and end of the game episode (760), the number of shots on the opponent's goal (42), and the number of players participating in the game episode (190) were recorded. The sample in the 11 vs. 11 format consists of 234 game episodes, in which the zones of the beginning and end of the game episode (936), the number of shots on the opponent's goal (31), and the number of players participating in the game episode (234) were recorded. In total, the observation sample corresponds to 2,193 coding elements and 424 game episodes in two game formats.

### 2.4 Variables

An item-coding template was developed to record the data. All input data relevant to each game were recorded using the Game Performance Assessment Tool (GPAI) (35). Each game was coded (36) by a team of observers specifically trained for this study and having 3 years of previous experience. Data quality control was performed using the consensus method (37) which assumes that observers discuss among themselves which category each unit of the coding element belongs to and reach agreement among themselves before recording them (31). Data control was also carried out by comparing the data recorded by observers in both formats (38)

The following elements were coded for each game Table 1, because they satisfy the conditions of exhaustive completeness and mutual exclusivity (39–42).

**Table 1:** Coding elements with codes.

Nr.	Elements	Codes and short description
1	Ball control	The observed team has possession of the ball (code BK); the opposing team has control of the ball (BKP); impossibility of observation (BKA).
2	Game episode	A game episode is considered the set of team actions while an observed team is in control of the ball (BIS); the ball is out of play (BNS)
3	Game episode start zone	The area where an observed team starts having possession of the ball. Confidence zone (left zone—Z1, center zone—Z2, right zone—Z3); forming zone (observed team half of field—Z4a, Z5a, Z6a; opposite team half of field Z4b, Z5b, Z6b); completion zone (Z7, Z8, Z9)
4	Game episode end zone	The area where an observed team loses possession of the ball and the opponent gains control. Confidence zone (left zone—Z1, center zone—Z2, right zone—Z3); forming zone (observed team half of field—Z4a, Z5a, Z6a; opposite team half of field Z4b, Z5b, Z6b); completion zone (Z7, Z8, Z9)
5	Shot at the opponent's goal	An observed team has a shot at the opponent's goal (S)
6	Changing the direction of the attack	It is a group tactical move that allows the ball to be moved from one longitudinal zone to the opposite one. Changing the direction of the attack using the long pass (SEG); Any movement of the ball from one side longitudinal zone to the opposite involving players located in the central zone (SEI)
7	Number of players	Number of players on the observed team involved in a single game episode (1.15)

### 2.5 Statistical analysis

The regular chi-square test compared zone use in different game formats. As a generalization of McNemar's chi-square test for related events (e.g., distribution of the beginning and end of an event) in a 2 × 2 table, Bowker's test (43) evaluated the distribution of the start and end of game episodes in a 3 × 3 table, considering three zones for the start and three zones for the end of episodes. We have applied an interactive calculation tool (44) for the chi-square test and the 'stats' package (45) in R for Bowker's test. The assessment of asymmetry (an indicator of a significant trend in data) represented the effect size (level of expression of the trend) for Bowker's test (43), and Cramer's V represented the effect size for the chi-square test. For both tests, 0.10, 0.30, and 0.50 were considered small, medium, and large effect sizes, respectively. Following Cohen (46), small effects refer to differences that need a thorough assessment, medium effects could be visible to “the naked eye”, and large effects are apparent. The relative involvement of the players in episodes under different game formats was compared with a nonparametric Mann–Whitney test as an alternative to the *t*-test.

## 3 Results

Table 2 presents the absolute frequencies of the start and end zones of the game episode. Absolute frequencies reflect the number of game episodes if their start and end were in one of the game zones. The overall distribution of the start and end frequencies of the episodes confirms the uneven distribution of the game episodes by the start and end zones. This was confirmed significantly (*p* < 0.001) by the values of Bowker's test for the related measurements (episode start and end) within the formats 11 vs. 11—*χ*^2^(3) = 129.97 and 9 vs. 9—*χ*^2^(3) = 149.44.

**Table 2:** Distribution of absolute zone frequencies at the beginning and end of a game episode in the 9 vs. 9 and 11 vs. 11 formats.

Zone of the field (for observed team)		Game format	The game episode start zone	The game episode end zone
Confidence zone		9 vs. 9	125	13
		11 vs. 11	106	24
Forming zone		9 vs. 9	55	50
		11 vs. 11	101	103
Completion zone		9 vs. 9	10	127
		11 vs. 11	27	107
Game format 9 vs. 9		The game episode start zone, number		
		Confidence	Forming	Completion
The game episode end zone	Confidence	12	1	0
	Forming	39	9	2
	Completion	74	45	8
Game format 11 vs. 11		The game episode start zone, number		
		Confidence	Forming	Completion
The game episode end zone	Confidence	20	3	1
	Forming	64	37	2
	Completion	22	61	24

The chi-square test was also used to compare the two-game formats if the 11 vs. 11 format represented the expected frequency distribution of the episodes. The test calculation revealed a significant deviation in the frequency distribution of the 9 vs. 9 format from the expected frequencies, *χ*^2^ (4) = 173.24, *p* < 0.001, representing a medium effect size, *V* = 0.45. However, in more than 20% of cells, the observed and expected frequencies were less than 5. This limited statistical conclusions and led to the grouping of individual episode categories, combining them into larger categories.

The first grouping was performed to assess the frequency of reaching the completion zone from the confidence zone in both game formats. When grouping observations, episodes that ended in the confidence or forming zone were combined (Table 3). As a result, it was found that there was a significant relationship between reaching the completion zone and the game format, *χ*^2^ (1) = 35.60, *p* < 0.001, *V* = 0.29 (close to the medium effect). This showed that game episodes starting in the confidence zone ended in the completion zone significantly more often in the 9 vs. 9 format (59.2% of episodes) than in the 11 vs. 11 format (20.8% of episodes).

**Table 3:** Distribution of reaching the completion zone from the confidence zone in the 9 vs. 9 and 11 vs. 11 formats.

Game format	The game episode ended in the completion zone, number	The game episode ended in another zone, number	Total, number
9 vs. 9	74 (59.2%)	51 (40.8%)	125 (100%)
11 vs. 11	22 (20.8%)	84 (79.2%)	106 (100%)

The second grouping was performed to assess whether to reach the completion zone of the forming zone. Episodes that ended in the forming zone or by leaving the confidence zone were aggregated (Table 4). It was found that there was a significant relationship between reaching the completion zone from the forming zone and the game format, *χ*^2^ (1) = 6.73, *p* = 0.010, *V* = 0.13 (small effect size). Game episodes that started in the forming zone ended in the completion zone significantly more often in the 9 vs. 9 format (81.8% of episodes) than in the 11 vs. 11 format (60.4% of episodes).

**Table 4:** Distribution of reaching the completion zone from the forming zone in the 9 vs. 9 and 11 vs. 11 formats.

Game format	The game episode ended in the completion zone, number	The game episode ended in another zone, number	Total, number
9 vs. 9	45 (81.8%)	10 (18.2%)	55 (100%)
11 vs. 11	61 (60.4%)	40 (39.6%)	101 (100%)

In step three, the grouping was performed to compare the frequencies of the game episodes that ended in the forming and completion zones to see if the beginning of the episode was in the confidence or forming zone (Table 5). This combined the number of ended episodes in the forming zone if the start was in the confidence or forming zone, as well as the number of ended episodes in the completion zone if the start was in the confidence or forming zone. A significant relationship was found between the game format and the endings of the episodes in the completion or forming zone, *χ*^2^ (1) = 23.51, *p* < 0.001, *V* = 0.24 (relatively small effect). Game episode endings in the completion zone were more common in the 9 vs. 9 format (71.3% of episodes) than in the 11 vs. 11 format (45.1% of episodes), while endings in the forming zone were more common in the 11 vs. 11 format (54.9%) than in the 9 vs. 9 format (28.7%).

**Table 5:** Distribution of end of a game episode in the completion or forming zone in 9 vs. 9 and 11 vs. 11 formats.

Game format	The game episode ended in the completion zone, number	The game episode ended in the forming zone, number	Total, Number
9 vs. 9	119 (71.3%)	48 (28.7%)	167 (100%)
11 vs. 11	83 (45.1%)	101 (54.9%)	184 (100%)

To characterize the effective action in the completion zone, the number of shots in the opponent's goal and other outcomes of the game episodes in two game formats (9 vs. 9 and 11 vs. 11) were evaluated. It should be noted that the relative frequency of effective actions (shots at the opponent's goal) in both formats was relatively similar (Table 6). The evaluation of the relationship between the frequency of effective actions and the game format confirmed this similarity, *χ*^2^ (1) = 0.46, *p* = 0.500, *V* = 0.03.

**Table 6:** Distribution of effective and ineffective actions in the completion zone by game format.

Game format	Effective action, number	Ineffective action, number	Total, number
9 vs. 9	42 (33%)	85 (67%)	127 (100%)
11 vs. 11	31 (29%)	76 (71%)	107 (100%)

**Figure 2:**
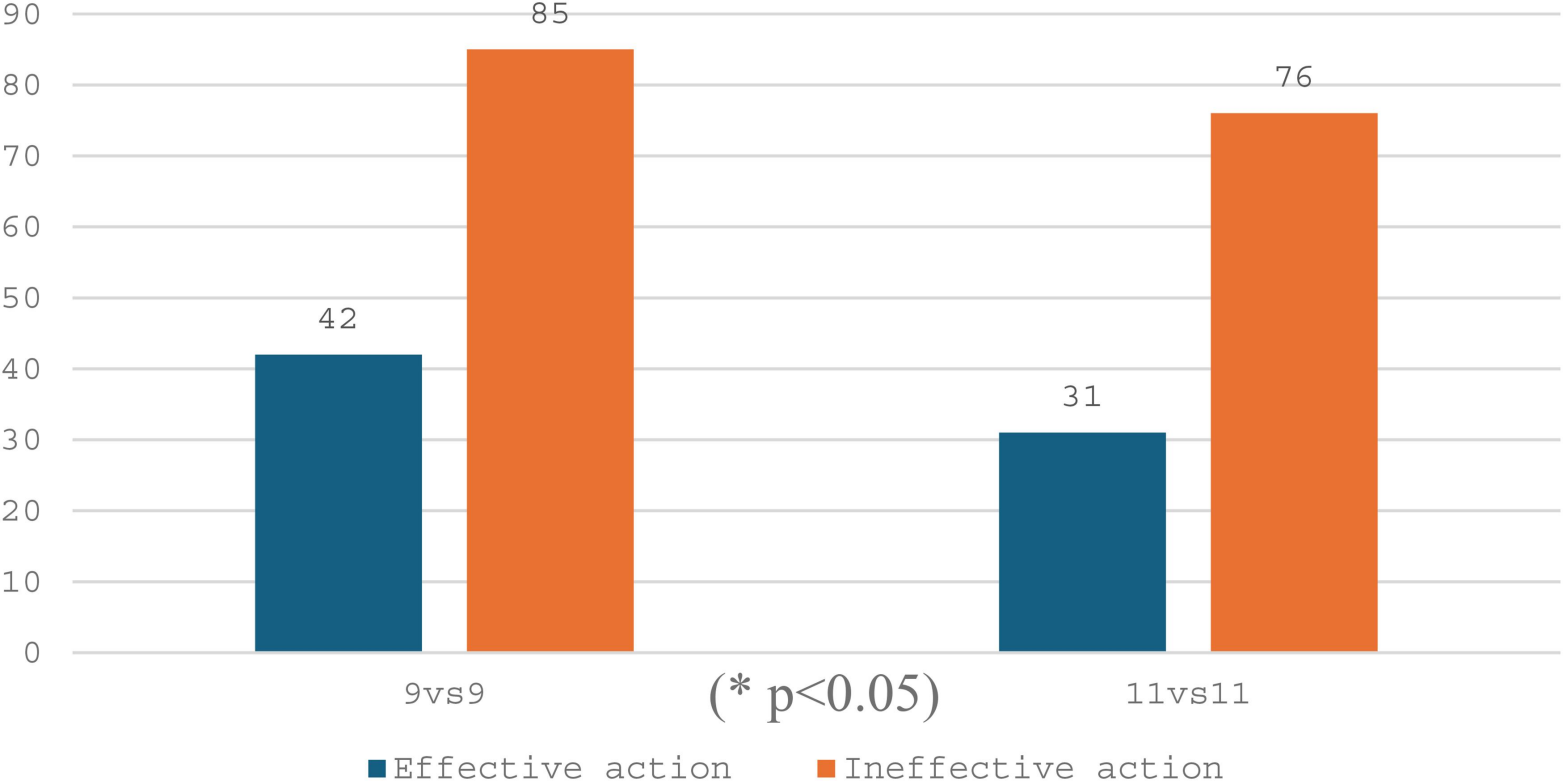
Distribution of absolute frequencies (number) of effective actions in the completion zone by game format.

At the same time, if we assume that the episodes of the game in the 11 vs. 11 format constitute the expected frequency during the game, then the frequency of the episodes observed in the 9 vs. 9 format was significantly higher, *χ*^2^ (1) = 4.97, *p* = 0.026, *V* = 0.11 (small effect size). Therefore, the format of the 9 vs. 9 game allows a significantly larger total number of game episodes than the format of 11 vs. 11—while leaving the proportion of effective actions relatively unchanged (Figure 2).

The results of the Bowker's test for related measurements (start and end of the game episode) did not show a significant trend in the use of longitudinal zones within the 11 vs. 11 format, *χ*^2^ (3) = 1.03, *p* = 0.795, and the 9 vs. 9 format, *χ*^2^ (3) = 0.21, *p* = 0.976. The effect sizes were 0.02 for the 11 vs. 11 format and 0.04 for the 9 vs. 9 format. These calculations confirmed that the game is symmetric in the transverse plane in both formats (Table 7).

**Table 7:** Distribution of absolute frequencies of longitudinal zones used at the beginning and end of a game episode in the 9 vs. 9 and 11 vs. 11 formats.

Longitudinal zone of the field (for observed team)		Game format	The game episode start zone	The game episode end zone
Left zone	9 vs. 9		43	36
	11 vs. 11		64	60
Center zone	9 vs. 9		98	102
	11 vs. 11		96	99
Right zone	9 vs. 9		49	52
	11 vs. 11		74	75
Game format 9 vs. 9		Game episodes start longitudinal zones, number		
		Left	Central	Right
Game episodes end longitudinal zones, number	Left	14	18	4
	Central	23	48	31
	Right	6	32	14
Game format 11 vs. 11		Game episodes start zone, number		
		Left	Central	Right
Game episodes end longitudinal zones, number	Left	24	27	9
	Central	30	44	25
	Right	10	25	40

A comparison of the total frequency of episodes between the formats 9 vs. 9 and 11 vs. 11 also did not show significant differences in use or change of a longitudinal zone, *χ*^2^ (4) = 1.37, *p* = 0.849, *V* = 0.04. Finishing and starting the episode in the same zone was relatively similar between the formats. There were 76 (or 40%) cases in the 9 vs. 9 format and 108 (46.2%) cases in the 11 vs. 11 format.

At the same time, in the 9 vs. 9 format, the team that controlled the ball and started the game episode in one of the side longitudinal zones of the field had a much greater chance of finishing the game episode in the central longitudinal zone 54 of 92 (58.7%) cases than in the 11 vs. 11 format with 55 of 138 (41.7%) cases, *χ*^2^ (1) = 7.86, *p* = 0.005, *V* = 0.18 (small effect size).

An additional analysis of the episodes demonstrated that the changing direction of the attack in the 9 vs. 9 format occurred in 50 cases (or 26.3% of all game episodes) and 16 cases (6.8%); in the 11 vs. 11 format. This difference was also significant, *χ*^2^ (1) = 33.58, *p* < 0.001, *V* = 0.28 (close to medium effect size).

The participation of players in-game episodes was compared, accounting for a relatively low number of episodes involving more than 10 players (Table 8). Cells of 11–15 players were merged for both formats. The results indicated that there were no significant differences between the distributions of involved players, *χ*2(10) = 12.59, *p* = 0.248, *V* = 0.05. It can be concluded that a smaller number of players in the 9 vs. 9 format did not lower the number of players involved in each game episode compared to the 11 vs. 11 format.

**Table 8:** A relative frequency of game episodes with different numbers of involved players under 9 vs. 9 and 11 vs. 11 formats.

Game format 9 vs. 9			Game format 11 vs. 11		
Number of players in the episode	Number of episodes	Relative frequency of episodes	Number of players in the episode	Number of episodes	Relative frequency of episodes
1	4	2.1%	1	16	6.8%
2	23	12.1%	2	58	24.8%
3	44	23.2%	3	64	27.4%
4	27	14.2%	4	30	12.8%
5	26	13.7%	5	19	8.1%
6	18	9.5%	6	18	7.7%
7	12	6.3%	7	9	3.8%
8	10	5.3%	8	5	2.1%
9	11	5.8%	9	6	2.6%
10	4	2.1%	10	5	2.1%
11	2	1.1%	11	1	0.4%
12	1	0.5%	12	0	0.0%
13	3	1.6%	13	1	0.4%
14	1	0.5%	14	0	0.0%
15	4	2.1%	15	2	0.9%

**Figure 3:**
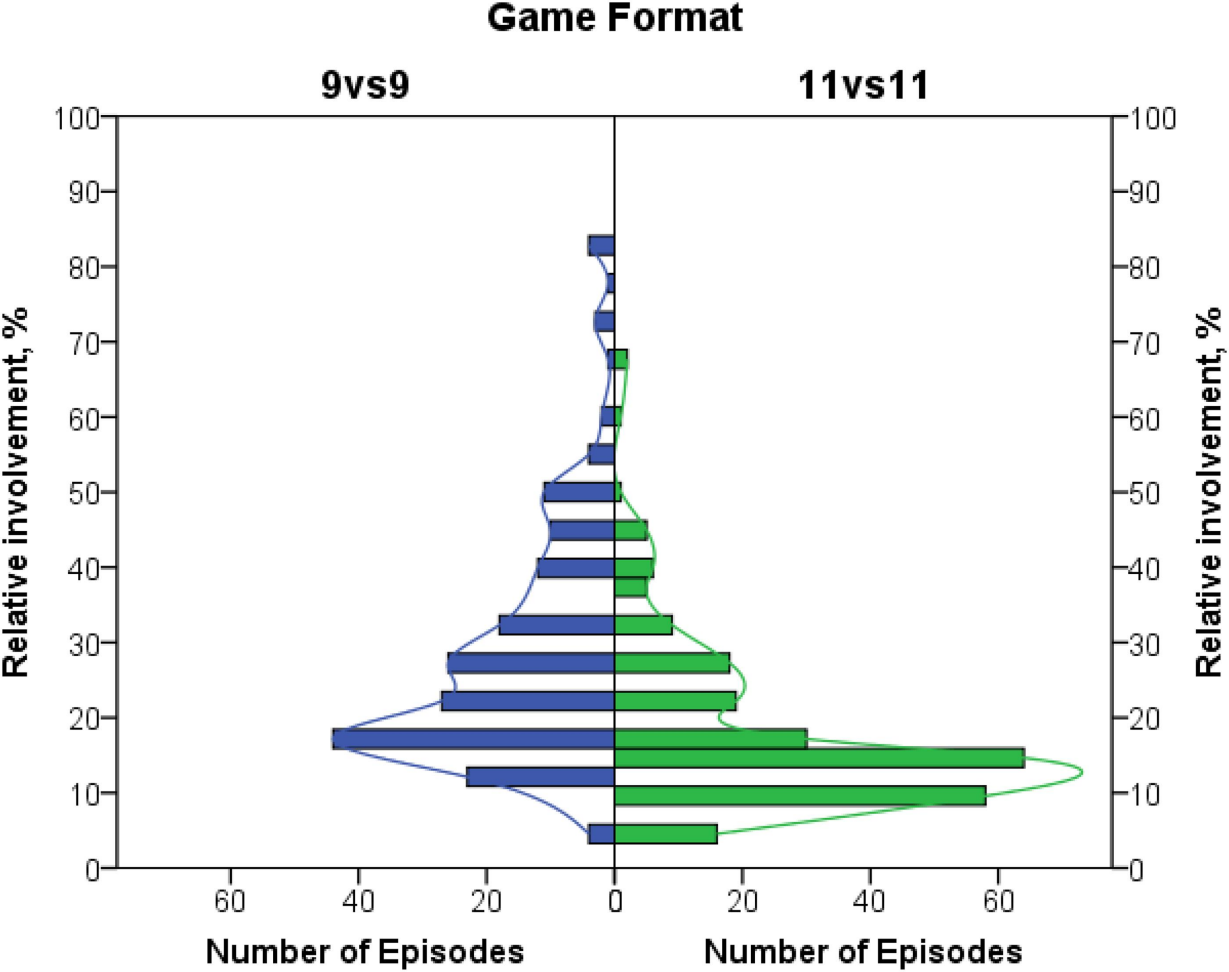
Distribution of relative proportions of involved players by game format.

Three players per game episode was the mode in both formats. At the same time, the total number of players differed between formats, and the proportions of players involved were compared between formats. Nonnormally distributed, they were compared in a nonparametric way and showed a higher proportion of players involved in each episode in the 9 vs. 9 format (Figure 3) than in the 11 vs. 11 format, *U* = 11,998.50, *p* < 0.001, effect size *r* = 0.40 (medium effect).

The median involvement of players in the 9 vs. 9 format was 22.2% vs. 13.6% in the 11 vs. 11 format. Therefore, the 9 vs. 9 format demonstrated a more intensive participation of the players in the game episodes.

## 4 Discussion

The main objective of this study was to identify trends in the use of playing areas and to investigate the performance of players in the completion zone in the age group U13 with varying numbers of players and sizes of field dimensions. The absolute frequencies of the zones of the beginning and end of game episodes in both formats confirm the unevenness of their distribution, as well as a significant shift in the frequency distribution of the 9 vs. 9 format from the expected frequencies of the 11 vs. 11 format, indicating the emergence of a tendency for players to spread out on the field; that is, the larger the field, the more the players are spread out on it, and less conditions for actions to create space for their teammates which are align with the results of (47, 48).

In line with Costa et al. (49), fewer players on a team were linked to faster transitions and more completion zone opportunities. Episodes starting in the confidence zone reached the completion zone more in 9 vs. 9 (59.2%) than 11 vs. 11 (20.8%), showing that smaller formats favor attacking success, similar to the study by (50).

Episodes from the forming zone ended in the completion zone more in 9 vs. 9 (81.8%) than 11 vs. 11 (60.4%), indicating larger formats favor ball control over sharp attacks (51). Overall, forming-to-forming or completion episodes were more frequent in 9 vs. 9 (71.3%) than 11 vs. 11 (45.1%), suggesting 9 vs. 9 players reach the completion zone faster and more effectively. In contrast, 11 vs. 11 players who aim for a less direct style of play, as the increase in the relative playing area leads to a gain in the number of defensive actions of players against the attacking team (52).

In addition, in both formats, the outcome of the game sequences remained largely constant in relation to the effectiveness of the actions in the completion zone (an effective action is a shot at the opponent's goal). The relatively similar frequencies of effective actions (33% for 9 vs. 9% and 29% for 11 vs. 11) and higher outcome of game sequences created in the 9 vs. 9 format, but with around the same efficiency of execution, may indicate that the skills that players possess transfer from the smaller to the larger format effectively, thus highlighting the importance of adaptive training methods across formats (53). The consistent ratio of effective to ineffective actions in both formats suggests that the scale of the game may not have a major impact on the quality of players' decision making in the completion area or when using a smaller game format (5 vs. 5 or 7 vs. 7), with the difference between successful actions being more substantial (54).

The results confirmed that the game formats in this age group cause significant differences in player positioning and movement during football matches (55). This finding suggests that the smaller playing area and the reduced number of players in the 9 vs. 9 game format can create more opportunities for players to reach the completion zone and take shots at the opponent's goal, which is also confirmed by the study (22). On the other hand, the 11 vs. 11 game format promotes greater stability (56) and the consistent spatial distribution of players on the field, indicating that the specific positions and roles of players are more static and predictable.

Although the 11 vs. 11 format has more players on the field, the median participation of players in the 9 vs. 9 format was 22.2%, compared to 13.6% in the 11 vs. 11 format, resulting in the 9 vs. 9 format seeing players more actively involved in play sequences that required ball movement at speeds and amplitudes like those seen in professional games (57) and thus promoting active learning (58).

It must be admitted that 11 vs. 11 game format might not provide an appropriate learning environment in the U13 age group (59), but frequent changes in the direction of attack (50 vs. 16 cases) in 9 vs. 9 format, as well as more successful penetration the central completion zone (113 vs. 92 cases), support nonlinear pedagogy in the developing individual game play and decision-making abilities (60).

The statistical significance of the impact of the game format on the development of game episodes also supports the idea that coaching methodologies should be adapted to the competition format (13). Moreover, for (A) tactical implications, coaches working with U13 players could benefit from focusing on individual and group tactical aspects of their game during the 9 vs. 9 format, as it encourages more action in critical areas of the playing field, completion, and confidence zones. Implementing drills and training matches that replicate the 9 vs. 9 format, quickly switching from attack to defense and vice versa, and bypassing the forming zone as quickly as possible, will help optimize training sessions for developing specific skills and abilities, as demonstrated precisely in critical areas of the playing field. When preparing players for the 11 vs. 11 game format, and because players tend to be more spread out on a larger playing field, it is important to include drills in training sessions that improve spatial awareness and positioning. This may consist of drills that require players to maintain optimal spacing while moving the ball efficiently. (B) Players’ developments, the study has identified clear differences between formats 9 vs. 9 and 11 vs. 11, within the patterns used in the different formats. In the 9 vs. 9 format, a player operates within one box to the other, gaining maximum game experience at his own or the opponent's goal. In the 11 vs. 11 format, teams tend to play more defensively, focusing on team interactions, and only rarely get the opportunity to go through the central zone and threaten the opponent's goal. (C) Despite limitations of the pilot study data by sample size and the design of the quasi-experiment in the form of friendly games, which might result in less accurate effect size estimates, given the exploratory stage and preliminary scope of the research, the future research should aim to involve larger sample sizes and conduct rigorous sample size calculations to increase statistical robustness. While the small sample size limits generalizability, this kind of research is essential to comprehend how the format of the game influences the long-term development trajectory of young football athletes. This study is one of the first quasi-experimental studies on the impact of game formats on shaping the long-term development of young football players through game patterns. Future research should be directed toward examining the impact of different game formats on each age group, assessing their sequencing and progression, and analyzing how all these aspects interact with a model of comprehensive long-term athlete development. Other variables, such as coaching styles, player motivation, and age-appropriate game contexts, need to be explored.

## 5 Conclusion

This research has highlighted distinct distinctions between the 9 vs. 9 and 11 vs. 11 formats, illustrating the mechanisms through which players can undergo development by engaging with the various structures inherent in these formats. In the 9 vs. 9 format, a player operated from box to box, gaining significant experience both at their own and the opponent's goal, frequently switching tactical roles between defense and attack. Conversely, in the 11 vs. 11 format, a player tended to adopt a more defensive stance, emphasizing team interactions with a more static tactical role on the field and rarely advancing through the central zone to threaten the opponent's goal. Moreover, the later this transition is made, the easier it will be for the coach to create a powerful team that is made up of strong football individuals.

## Data Availability

The datasets presented in this study can be found in online repositories. The names of the repository/repositories and accession number(s) can be found below: https://failiem.lv/u/xpms4ccyuh. Further inquiries can be directed to the corresponding author.
